# The mediating role of impulsivity in the relationship between suicidal behaviour and early traumatic experiences in depressed subjects

**DOI:** 10.1192/j.eurpsy.2021.1556

**Published:** 2021-08-13

**Authors:** F. Dal Santo, J.J. Carballo, Á. Velasco, L. Jiménez-Treviño, J. Rodríguez-Revuelta, C. Martínez-Cao, I. Irene Caro-Cañizares, L. De La Fuente-Tomás, I. Menéndez-Miranda, L. González-Blanco, M.P. García-Portilla, J. Bobes, P.A. Saiz

**Affiliations:** 1 Department Of Psychiatry, University of Oviedo, Oviedo, Spain; 2 Department Of Psychiatry, Hospital General Universitario Gregorio Marañón, Madrid, Spain; 3 Department Of Psychiatry, Fundación Jiménez Díaz Hospital, Madrid, Spain; 4 Psychiatry, University of Oviedo, Oviedo, Spain

**Keywords:** Stressful Life Events, childhood trauma, suicidal behaviour, Impulsivity

## Abstract

**Introduction:**

Depressed patients with early traumatic experiences may represent a clinically distinct subtype with worse clinical outcome. Since early traumatic experiences alter the development of systems that regulate the stress response, certain personality features may influence coping strategies, putting individuals with depression and a history of early traumatic experiences at greater risk of suicidal behaviour.

**Objectives:**

To determine whether impulsivity mediates the relationship between early traumatic experiences and suicidal behaviour in patients with major depressive disorder (MDD).

**Methods:**

The sample included 190 patients [mean age (SD)=53.71 (10.37); females: 66.3%], with current MDD. The Childhood Trauma Questionnaire-Short Form (CTQ-SF), the List of Threatening Experiences (LTE), and the Barratt Impulsiveness Scale-11 (BIS-11) were used to assess childhood and adulthood adverse life events and impulsivity. We developed mediation models by bootstrap sampling methods.

**Results:**

81 (42.6%) patients had previous suicide attempts (SA). CTQ-SF-Total and BIS-11-Total scores were higher in MDD patients with previous SA. Correlation analyses revealed significant correlations between the CTQ-SF-Total and BIS-11-Total, CTQ-SF-Total and HDRS-Total, and BIS-11-Total and HDRS-Total scores. Regression models found that CTQ-SF-Total, BIS-11-Total, and HDRS-Total scores were associated with SA. Mediation analyses further revealed the association between CTQ-SF-Total and SA was mediated by the indirect effect of the BIS-11-Total score (b=0.007, 95% CI=0.001, 0.015), after controlling for sex, HDRS-Total, and LTE-Total.

**Conclusions:**

Impulsivity could mediate the influence of childhood trauma on suicidal behaviour. This will help understand the role of risk factors in suicidal behaviour and aid in the development of prevention interventions focused on modifiable mediators when risk factors are non-modifiable.

